# Haptic perception of force magnitude and its relation to postural arm dynamics in 3D

**DOI:** 10.1038/srep18004

**Published:** 2015-12-08

**Authors:** Femke E. van Beek, Wouter M. Bergmann Tiest, Winfred Mugge, Astrid M. L. Kappers

**Affiliations:** 1 MOVE Research Institute, Department of Human Movement Sciences, VU University Amsterdam, The Netherlands

## Abstract

In a previous study, we found the perception of force magnitude to be anisotropic in the horizontal plane. In the current study, we investigated this anisotropy in three dimensional space. In addition, we tested our previous hypothesis that the perceptual anisotropy was directly related to anisotropies in arm dynamics. In experiment 1, static force magnitude perception was studied using a free magnitude estimation paradigm. This experiment revealed a significant and consistent anisotropy in force magnitude perception, with forces exerted perpendicular to the line between hand and shoulder being perceived as 50% larger than forces exerted along this line. In experiment 2, postural arm dynamics were measured using stochastic position perturbations exerted by a haptic device and quantified through system identification. By fitting a mass-damper-spring model to the data, the stiffness, damping and inertia parameters could be characterized in all the directions in which perception was also measured. These results show that none of the arm dynamics parameters were oriented either exactly perpendicular or parallel to the perceptual anisotropy. This means that endpoint stiffness, damping or inertia alone cannot explain the consistent anisotropy in force magnitude perception.

The use of tele-operation systems in dangerous, remote and small-scale environments is increasing. To provide the operator with a more complete picture of the environment at the slave side, providing haptic feedback in addition to visual feedback can be useful^1^. To understand how to design force feedback that is intuitive for the user, knowledge on human force perception is important. Our previous study^2^ has shown that the perception of force magnitude in 2D (in the horizontal plane) is significantly and systematically anisotropic. This anisotropy fitted quite well with arm dynamics data from literature^3^,^4^. Nonetheless, important questions still remained unanswered. How is force magnitude perception distributed outside of the horizontal plane? And is this distribution indeed related to arm dynamics? Force perception in 3D will be anisotropic, given that it is anisotropic in the horizontal plane. However, the orientation and shape of the complete anisotropy cannot be predicted from the 2D data. The direction in which forces are perceived as being the largest could, for instance, already have been found in the 2D measurements in the horizontal plane, or it could be more vertically oriented. Furthermore, the shape of the 3D anisotropy could be very elongated or more spherical. From a fundamental point of view, it is interesting to obtain a complete picture of the orientation and shape of the anisotropy in force magnitude perception, as the perception of forces is crucial in the interaction of humans with their environment. Moreover, it is still unknown if there is a link between arm dynamics and force magnitude perception^5^. So, our aim is to determine if this link indeed exists and therefore if the anisotropy in arm impedance might explain the perceptual anisotropy, by assessing if both data sets are oriented perpendicularly. Our hypothesis is that the direction in which forces are perceived as being the largest is perpendicular to the direction in which arm dynamics parameters are the largest, as the latter is the direction in which it is easier to resist the force. To this end, a good description of the shapes of both data sets in 3D is needed. So, in summary, the previous study was extended in two ways: 1) perception was measured in three dimensions and 2) postural arm dynamics were measured to be able to directly relate arm dynamics and force perception.

Force magnitude perception in directions other than that of gravity has mainly been studied in experiments investigating discrimination thresholds^6^,^7^,^8^,^9^. In the current article, we focus on the effect of direction on the perceived magnitude of the force, which has only been studied by two research groups^8^,^10^. Dorjgotov *et al.*^8^ asked participants to discriminate between the magnitude of a reference force, exerted along the dorso-ventral axis towards the participant, and a test force exerted along one of the other cardinal axes. Forces were exerted through a haptic device with a ball-shaped gimbal, which participants enclosed with the whole hand. For all the test axes, a significant perceptual difference with the reference axis was found, indicating that a force towards the participant was perceived as being smaller than a force exerted along any of the test axes. The bias between test and reference axis was comparable for all test axes, which suggests that there was no perceptual difference between the test axes. However, the test axes were not compared directly and no other directions than the cardinal axes were tested. Tanaka and Tsuji^10^ found elliptical anisotropies in a study using large forces in a similar task: a comparison between a tangential reference force and test forces along 8 directions in the horizontal plane. A more direct way to investigate the distribution of force magnitude perception is by using a free magnitude estimation paradigm, which determines the relation between physical and perceived magnitude^11^. We used this method in our previous study^2^ to assess force magnitude perception in several non-cardinal directions in the horizontal plane, in which we found an elliptically shaped distortion. In the present study, our main aim is to investigate the shape of the perceptual anisotropy in 3D, by extending our perceptual measurements to directions outside of the horizontal plane. To assess if anisotropies in arm dynamics are directly related to the shape of the perceptual anisotropy and could therefore be an explanation for it, we also measured arm dynamics.

Research into arm dynamics started by using step-like position perturbations to measure the stiffness, damping and inertia of the arm^3^,^12^,^13^,^14^,^15^,^16^,^17^. Another approach, which has gained influence over the last years, is the use of stochastic perturbations to determine arm impedance^18^,^19^. In this approach, force (or position) is imposed as a continuous and unpredictable perturbation on the arm, while position (or force) is measured. The transfer function between the position and force signals describes arm admittance (or impedance), while the only prior assumption is linearity. Afterwards, models can be fitted to the measured transfer function to describe it (see e.g. van der Helm *et al.*^20^); a mass-damper-spring model seems to be a fair approximation for small arm displacements, when participants are asked not to intervene with the perturbations^21^. In several studies, correlations between arm dynamics and motor task execution have been found (e.g.^4^,^22^,^23^,^24^,^25^,^26^). For instance, Trumbower *et al.*^25^ showed that when participants are free to choose a posture in a tracking task in an unstable environment, they select a posture that shifts maximum arm impedance towards the direction of instability. Sabes *et al.*^22^ describe that humans tend to adjust movements around obstacles by rotating the inertia ellipsoid in such a way that the inertia of the arm is maximal at the point where collision with the object is most likely. Cos *et al.*^26^ showed that humans choose a biomechanically optimal path to a target, when choosing between two paths that do not differ in any other aspect. Most strikingly, humans are able to make this decision within 200 milliseconds. A link between arm impedance and motor task execution is not surprising, as motor task execution is obviously influenced by arm dynamics. By measuring arm dynamics and comparing them to perceptual data, we investigate the presence of such a link between arm dynamics and a perceptual task.

The main aim of this study was to investigate the shape of the anisotropy in force magnitude perception in 3D. Furthermore, we measured postural arm dynamics - the impedance of the arm in a given posture - to investigate its correlation with the perceptual data. In experiment 1, force perception was measured using a free magnitude estimation paradigm. In experiment 2, postural arm dynamics were measured using a variation of a method described previously, using stochastic position perturbations^4^,^25^,^27^. Our data provide information about anisotropies in human force perception and arm dynamics and test the hypothesis that anisotropies in force magnitude perception are directly related to anisotropies in arm dynamics. If force perception could be predicted by modeling arm dynamics, predictions concerning, for instance, the effect of different postures could be verified. So, the application of this fundamental knowledge could contribute to designing force-feedback algorithms for tele-operation systems that correct for anisotropies in human force perception.

## Results

### Experiment 1: Perception

**Figure 1:**
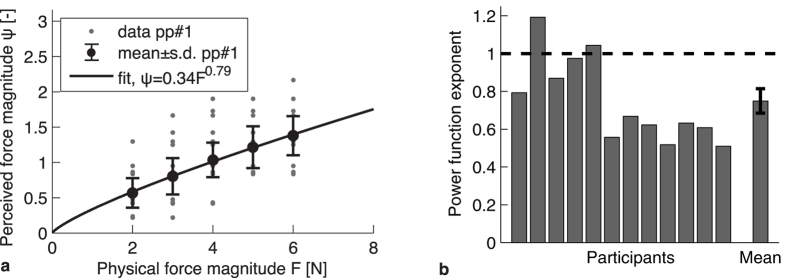
Overview of the perceptual data, showing the relation between physical and perceived force magnitude, measured using free magnitude estimation. (**a**) Typical example of force magnitude perception data of one participant, for all directions together. The small grey dots show individual measurements, while the thick black dots with error bars show the mean per physical force magnitude ± standard deviation. The black line shows the fit of the power function, which describes the relation between physical force magnitude (*F*) and mean perceived force magnitude (*ψ*). The fit was used to normalize the data. (**b**) Exponents of the fitted power functions, per participant and mean ± standard error over participants. Note that on average, the power function exponents differ from 1, indicating a non-linear relationship between physical and perceived force magnitude.

In experiment 1, participants verbally reported the perceived magnitude of force stimuli, which could be exerted in 26 directions and with 5 different magnitudes. A typical example of force perception data is shown in Fig. 1a. For each physical force magnitude, some spread in the perceived force magnitudes can be seen (the spread of the thin gray dots around the thick black dots in Fig. 1a), which had a standard deviation of 0.30 on average across participants and physical force magnitudes. Power functions were fitted to the perceptual data of each participant separately, averaged per physical force magnitude. Since a power function can only describe the mean variation caused by the independent variable and not the noise around the mean^11^, the fit was performed on the mean values per physical force magnitude. An example of such a fit is also shown in Fig. 1a. The goodness-of-fit was excellent, with *R*^2^ values being greater than 0.98 for each participant.

In Fig. 1b, the fitted power function exponents are shown for each participant. For 10 of the 12 participants, the exponent was lower than 1, suggesting a non-linear relationship between physical and perceived force magnitude. A one-sample *t*-test on the exponents showed that they indeed differed significantly from 1 (*t*_11_ = −3.9, *p* = 0.003). Since the relation between physical and perceived force magnitude was non-linear, normalizing the data across physical force magnitudes could not be done by dividing the perceived magnitude by the physical magnitude. To still be able to assess the influence of direction, irrespective of physical force magnitude, normalization was done by calculating the fitted perceived force magnitude values per physical force magnitude according to the fitted power function. The actually perceived force magnitude values were divided by these calculated fit values to normalize the data.

On these normalized data, a repeated measures ANOVA was performed, which showed a significant effect of force direction on perceived force magnitude (*F*_25,275_ = 10.6, *p* < 0.001). This means that force magnitude perception is indeed anisotropic in 3D. To assess if the pattern of the perceptual anisotropy was different between the three measurement sessions or between the five physical force magnitudes, the interactions between session and force direction and between physical force magnitude and force direction were also tested in a repeated measures ANOVA. These interactions were not significant (*F*_1.1,60_ = 1.31, *p* = 0.27 and *F*_7.3,80_ = 1.43, *p* = 0.20, respectively), indicating that the patterns did not differ between sessions and physical force magnitudes. This shows, in retrospect, that averaging over sessions and physical force magnitudes did not influence the results.

**Figure 2:**
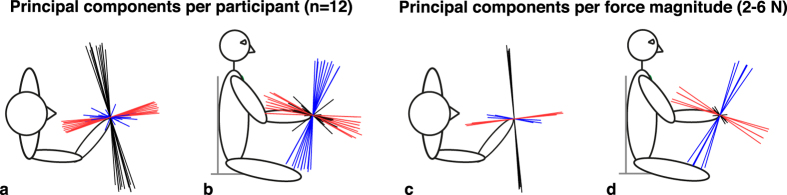
Perceptual data, showing the three axes of the Principal Component Analysis (PCA) per participant and per physical force magnitude. Black: first component, blue: second component, red: third component. Top (**a**) and side view (**b**), with each vector corresponding to one participant. Top (**c**) and side view (**d**), with each vector corresponding to one physical force magnitude, while the data were averaged over participants. The first component is considerably larger than the second and third component, resulting in eccentricities (i.e. the ratio between the eigenvalues of the third and first component of the PCA) that were well below 1. The orientation and size of the components are consistent over participants and physical force magnitudes. Together, this illustrates the consistent anisotropy in force magnitude perception.

A Principal Component Analysis (PCA) was performed to assess the orientation and the shape of the perceptual data set. An overview of the principal components is given in Fig. 2, which shows a striking similarity across participants and physical force magnitudes. The analyses showed that the ratio between the smallest and the largest value (i.e. the ratio between the eigenvalues of the third and first component of the PCA), which we will call eccentricity, was 0.66 ± 0.02 (mean ± standard error). The similarity of the orientation of the principal components and the eccentricity being considerably smaller than 1 together indicate a systematic distortion in force magnitude perception.

### Experiment 2: Arm dynamics

**Figure 3:**
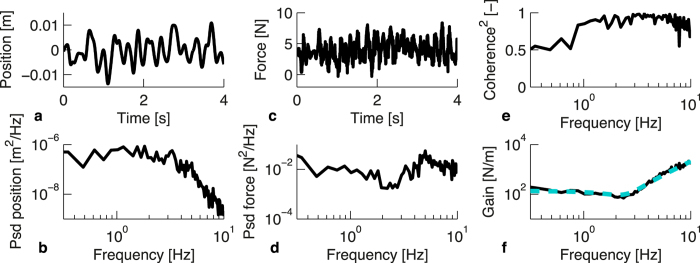
Typical example of force and position data of one trial of one participant in the arm dynamics measurements. (**a**,**b**) Time and frequency domain representation of the position input signal, as measured using Optotrak markers at the end effector of the haptic device. Only a few seconds of the time domain representations are shown. (**c**,**d**) Time and frequency domain representation of the force signal, as measured with the force sensor of the HapticMaster. (**e**) Squared coherence of the position and force data. (**f**) Frequency Response Function based on the ratio of the spectra in b and d. The dashed blue line shows the fit that was made using the mass-damper-spring model (see Equation 4). For this trial, the model yielded the following values: *M* = 0.554 kg, *B* = 6.45 Ns/m, *K* = 135 N/m.

**Figure 4:**
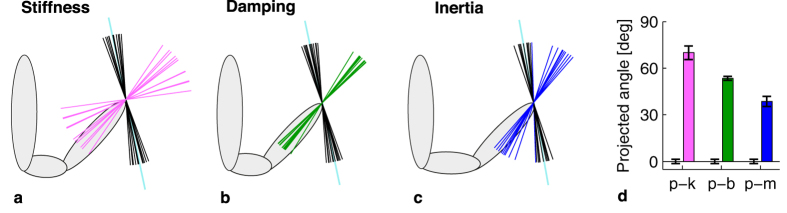
Comparison of the 2D projections of the first component of the PCA of perceptual data and the first component of the PCA of the three arm dynamics parameters. Each subplot shows a projection onto a slightly different plane, as the plane is determined by the mean vector of the perceptual data and the mean vector of the arm dynamics parameter of interest. Each line shows the orientation of the first component of the PCA of the data of one participant. All vectors are unit vectors. The light gray shapes show the projection of shoulders, upper and lower arm of a participant in the experimental posture. (**a**) Perception (black) and stiffness (pink) vectors. (**b**) Perception (black) and damping (green) vectors. (**c**) Perception (black) and inertia (blue) vectors. (**d**) Projected angle of each of the data sets shown in panel a–c, with respect to the light blue axis (which is defined by the mean of the perceptual data set, which is obviously 0 in this definition). Note that none of the arm dynamics parameters are oriented perpendicular or parallel (i.e. 90 or 0°) to the perceptual data.

In experiment 2, postural arm dynamics data were recorded at end-point level along the same 26 directions that were used in experiment 1. In Fig. 3, example data of one trial and all steps in the analysis of these data are shown. The analysis yielded values for arm stiffness, damping and inertia, for each participant and each direction. The consistency of the fit values across participants was determined by calculating the standard error of the values across participants per direction, after which these values were averaged across directions, resulting in an indication of the mean variation across participants per parameter. These calculations yielded a standard error of 22% for stiffness, 14% for damping and 13% for inertia. The average *R*^2^ over all participants and directions was 0.77 ± 0.03 (mean ± standard error). The analytical steps are described in more detail in the ‘Methods’ section. A repeated measures ANOVA on the stiffness, damping and inertia values showed that there was a significant effect of direction on all parameters (all *F*_25,275_ > 10.6, all *p* < 0.001). A PCA was performed on all three arm dynamics parameters for each participant, to reveal the orientation and shape of the data sets. These analyses yielded eccentricities of 0.73 ± 0.02 for stiffness, 0.51 ± 0.02 for damping, and 0.56 ± 0.02 for inertia (mean ± standard error). Clearly, all eccentricities are considerably smaller than 1, as the mean values are all more than 10 standard errors below 1. This confirms the effect of direction on all arm dynamics parameters, which means that there is an anisotropy in the arm dynamics parameters. The next question is if the anisotropies in arm dynamics data are related to the anisotropy in the perceptual data, i.e. if the perceptual data set is oriented perpendicular or parallel to one of the arm dynamics data sets. Experiment 1 and 2 were performed with different participants, so a comparison between the vectors on participant level was not possible. Instead, the perceptual vectors and the arm dynamics vectors were averaged to compare their mean orientation. To statistically compare the data sets, the orientation of each vector for each participant needed to be calculated, preferably using a single measure. To do this for each combination of perceptual data and one of the arm dynamics parameters, the vectors of both data sets were projected onto a plane spanned by the mean vectors of both data sets. So, to compare the perceptual and stiffness data, for instance, all stiffness and perception vectors were projected onto the plane spanned by the mean perception and the mean stiffness vector. The projections of all combinations of data sets are shown in Fig. 4a–c. For all combinations, the planes of projection were close to the plane spanned by the first and third component of the PCA on the perceptual data (see Fig. 2 for the PCA on the perceptual data). The angle between the projected data sets was 70 ± 5° for perception and stiffness, 54 ± 2° for perception and damping, and 39 ± 4° for perception and inertia (mean ± standard error.). To assess if these angles differed significantly from 0 or 90°, 2-sample *t*-tests with a predicted mean of respectively 0 and 90° were performed for each combination. If the data sets were oriented parallel (0°), this would mean that the direction of largest perception and arm dynamics values coincided, while a perpendicular orientation (90°) would mean that the direction of the smallest perceptual values coincided with the largest arm dynamics values.

However, the angles between the projected arm dynamics and perceptual data were significantly different from 0° (perception-stiffness: *t*_14_ = 15, *p* < 0.001, perception-damping: *t*_22_ = 28, *p* < 0.001, perception-inertia *t*_22_ = 11, *p* < 0.001) and from 90° (perception-stiffness: *t*_14_ = −4.4, *p* = 0.004, perception-damping: *t*_22_ = −19, *p* < 0.001, perception-inertia *t*_22_ = −14, *p* < 0.001). An overview of the angles between the projected axes is given in Fig. 4d.

## Discussion

In this study, we found systematic anisotropies in force magnitude perception and in arm dynamics in 3D. These anisotropies were consistent over participants for both experiments. However, the anisotropies in arm stiffness, damping and inertia were not oriented perpendicular or parallel to the perceptual anisotropies. The significant anisotropy in force magnitude perception in 3D is consistent with the anisotropy that we found in the horizontal plane in our previous study^2^. In three dimensions, the shape of this distortion is somewhat cigar-like, with its major axis oriented at a small angle with the transversal axis, while the minor axis is oriented approximately along the line between hand and shoulder (see Fig. 2). So, both the major and the minor axis lie close to the horizontal plane. On average, the perceptual eccentricity was 0.66, which means that a force exerted along the major axis was perceived as being 50% larger than when it would have been exerted along the minor axis (since 1/0.66 ≈ 1.5). The orientation and shape of the anisotropy was similar for all participants and for the five different force magnitudes that were used (2–6 N), which shows the consistency of the effect. Interestingly, our previous experiment^2^ was performed with a different setup with a different handle, which was a tube-shaped handle that was grasped in a power grip, while the current setup had a ball-shaped handle. Apparently, this did not have a significant influence on the perceptual results. The knowledge that force perception is anisotropic could be used in, for instance, force-feedback devices. In these devices, the magnitude of force feedback could be adjusted according to the anisotropies, which would result in an isotropic perception of force. In everyday life, we apparently correct for the anisotropic nature of force perception in motor task execution, since we interact naturally with objects in many configurations. However, when force-feedback is used in a haptic guidance-type of feedback^1^, the forces are not directly coupled to the environment anymore, but they are used to provide additional information. For instance, when haptic guidance along a desired movement trajectory is provided, it might be beneficial to provide users with perceptually consistent, rather than physically veridical force feedback, to make sure that the level of guidance is perceptually equal along different directions. Moreover, although humans may be able to correct for perceptual anisotropies, it does not necessarily mean that physically veridical feedback is optimal. In critical situations, conflicts between modalities might make a difference, for instance by increasing reaction times. An attempt to incorporate force perception anisotropies, inspired by fundamental work on perception of force and position, has been made in a haptic feedback device for kinesthetic training^5^.

The measurements of arm dynamics also yielded consistent results over participants, shown by the consistency in anisotropy orientation and by the relatively small variation in absolute values across participants. The orientation of the anisotropies in stiffness, damping and inertia found in our study were comparable to anisotropies found for 3D measurements of postural arm dynamics^4^,^21^. In the perceptual task, participants were asked to resist a force with a gradual ramp, which only resulted in limited movements around the start position, especially since trials with substantial movement were rejected. Since the movements were small, we compared our perceptual data to postural impedances. Our postural impedance values, measured in Experiment 2 using small perturbations around a given posture, were substantially higher than values found in the literature for tasks in which movement to a target position is required^28^. It is therefore likely that our measurements were dominated by contributions of short-range impedance^29^,^30^. In specific perturbation directions the rotations may have been sufficiently large to exceed the domain of short-range stiffness. Nonetheless, our absolute arm dynamics values were comparable to values found in other studies measuring postural arm dynamics^31^,^32^.

The arm dynamics that we measured were likely composed of intrinsic muscle properties and reflexive feedback^28^,^32^,^33^,^34^. These components were likely also present during the perceptual measurements. By using a one-dimensional approach for the arm dynamics measurements, we measured the anisotropies in a situation comparable to the perceptual measurements, as discussed in more detail in the ‘Methods’ section. To ensure that the contribution of all parameters was comparable in both experiments, we controlled the parameters that we regarded as the most important parameters influencing the orientation of arm dynamics: posture and task instruction. The same joint angles were used in both experiments, as posture is a very important determinant in the orientation of the stiffness, damping and inertia anisotropies^3^,^35^. Our task instruction was also aimed at keeping the situation comparable in both experiments. Firstly, we instructed participants to exert a constant force, using feedback of their force on a screen. Voluntary force generation is known to influence particularly the stiffness parameter^36^. Secondly, we instructed participants to not intervene with the perturbations. This instruction was meant to minimize the contribution of voluntary control and reflexive feedback. In the next paragraphs, both aspects of the instruction will be discussed in more detail.

The instruction ‘keep exerting this force’ was used to keep the contribution of voluntary force comparable in both experiments, because it has been found that voluntary force influences particularly the stiffness parameter^31^,^37^. Perreault *et al.*^36^ have shown that the orientation and area of stiffness ellipses measured in the horizontal plane are affected by the level of voluntary force. However, even the lowest voluntary force that they used, around 12 N, was much higher than the voluntary force used in our current experiment (4 N), while the effects on ellipse orientation that they reported were most prominent at very high forces (up to 72 N). Dolan *et al.*^14^ found no differences between the orientation of stiffness ellipses measured using a voluntary force of 1 N and those measured during a relax task. Furthermore, Krutky *et al.*^4^ showed that when participants interacted with environments that were unstable along one of the cardinal axes, and therefore could have induced stiffness adaptations in these directions, there was no difference between the orientations of the stiffness ellipsoids measured in 3D in the different unstable environments. An increase in overall ellipsoid size was found for stiffness, but for damping and inertia no changes in ellipsoid orientation or size were reported. Even when participants are asked to actively change the orientation of their stiffness ellipse by using visual feedback, they are only able to do so to a small extent when they concurrently have to exert a voluntary force^31^. So, we believe that the main difference between arm dynamics parameters that we would have measured if we would have used a true relax task and the parameters that we measured in our current experiment is a difference in size and not in orientation of the parameters. Since we are only interested in the orientation of the parameters and not in the absolute values, we consider the addition of a voluntary force to the arm dynamics measurement to be a cautious addition.

The instruction ‘do not intervene with the perturbations’ was used to minimize the contribution of voluntary control and reflexive feedback. In experiment 1, intrinsic muscle properties and reflexive feedback undoubtedly both contributed to the total impedance of the arm^28^,^32^,^33^,^34^, so they were also measured together in experiment 2. EMG signals were not recorded, so we could not quantify the contribution of the reflexive component directly. It would have been an option to include reflexive feedback in our model of the system^38^. However, as reflected by the good fits with the mass-damper-spring model without reflexive feedback, the contribution of reflexive feedback was small in our particular task^38^. Apparently, the participants minimized responses to the position disturbances following their task instruction. In position tasks (e.g. Pruszynski *et al.*^39^) or tasks involving movements to a target position (e.g. Kurtzer *et al.*^40^) reflexive feedback does substantially contribute to arm impedance, but its contribution is small in tasks in which participants are asked not to intervene with the disturbances (e.g. de Vlugt *et al.*^33^ and Schouten *et al.*^34^). Forbes *et al.*^32^ show that when participants are asked to increase their arm stiffness based on feedback of their EMG signals, they mainly increase their intrinsic stiffness, while hardly increasing their reflexive stiffness. In our experiment, we essentially do the same by asking participants to keep exerting a force (which corresponds to the required EMG level) and to not intervene with the perturbations (which corresponds to the small contribution of reflexive feedback). So, we used intrinsic parameters only to represent the human arm, because this is a simple and robust model, with minimal redundancy, while it is sufficient to answer our research question and captures the most important impedance properties. The adequate goodness-of-fit values and the consistency of the results across participants show that this model does provide a proper description of the measured arm impedance. Moreover, we again point out the small magnitude of the voluntary force in this experiment, making the task more closely resemble a true relax task, in which studies report minimal reflexive feedback, than a position task, in which studies do report considerable reflexive feedback^33^,^34^.

Our data indicate that stiffness, damping or inertia alone cannot explain the anisotropy in force magnitude perception, since none of the arm dynamics anisotropies were oriented perpendicular or parallel to the perceptual anisotropy, while all anisotropies were consistent over participants. However, this does not mean that there is no relation between force perception and arm dynamics. Because of the static nature of the perception task, we think that stiffness is the most likely candidate of the arm dynamics parameters to be involved. Stiffness was also the parameter that was the closest to perpendicular to the perceptual data set (70°). A perpendicular orientation of the data sets would have supported the intuitively logical explanation that large stiffnesses coincide with smaller perceived forces. However, the significant deviation from 90° shows that endpoint stiffness alone cannot be the parameter causing the consistent anisotropy in force magnitude perception. An alternative explanation could be sought in the simplicity of the model of the arm. One important assumption is that the whole arm acts as a single unit and no bi-articular muscles are involved, which is obviously a simplification. Bi-articular muscles cause joints to be coupled, which makes the prediction of stiffness at endpoint level more complex, because bi-articular muscles can exert force in different directions and with different magnitude, depending on which other muscles are activated concurrently^41^,^42^. This coupling has been shown to cause a directionally selective increase in stiffness when making movements in unstable environments^23^, although Perreault *et al.*^36^ report that bi-articular muscles play a smaller role in the total endpoint stiffness than mono-articular muscles do during measurements of postural arm dynamics. Speculating on this topic, we suggest that if bi-articular muscles indeed played a role in our measurements, then this could have resulted in an irregular shape of the stiffness distribution. If the shape of the distribution would be irregular and would, for instance, have multiple ‘major axes’, the major axis determined with a PCA does not necessarily describe the direction of the largest values. This could mean that the actual orientations of the largest arm stiffness values were somewhat different than the ones found in our approach. Another aspect that might be important for the anisotropy in force perception is the contribution of different types of information to the total perceptual result, such as information from muscle spindles, tactual information and the efference copy. For instance, producing a force yields a smaller estimation of its magnitude than perceiving a force does, which is thought to be a result of the efference copy in force production^43^. What the effect of these speculations would be on the assessment of the relation between arm dynamics and force perception, remains to be investigated.

Concluding, we found that force magnitude perception is anisotropic in 3D. This anisotropy was not directly related to arm stiffness, damping or inertia, when modeling the human arm as a single unit. A more detailed model of the arm, possibly including bi-articular muscles, might be needed to understand the nature of the anisotropy in force magnitude perception.

## Methods

### Participants

In both experiments, twelve right-handed (assessed using the Coren-test for handedness^44^) participants took part. In experiment 1, 4 males and 8 females participated, aged 23 ± 3 years (mean ± standard deviation), height 1.79 ± 0.09 m, with no known neurological disorders. In experiment 2, a new group of twelve right-handed people participated, consisting of 4 males and 8 females, aged 29 ± 3 years, height 1.73 ± 0.09 m. All participants gave written informed consent to participate in the study and were naive to the purpose of the experiment. For experiment 1, they received a compensation of 8 euros per hour. Prior to the experiments, they were given written and oral instructions on how to perform the experiment. From each participant, the following measures were recorded: height, arm span, shoulder-to-shoulder length, upper arm length and lower arm length. Both experiments were approved by the Ethics Committee of the Faculty of Human Movement Sciences (ECB) and were carried out in accordance with the approved guidelines.

### Setup

**Figure 5:**
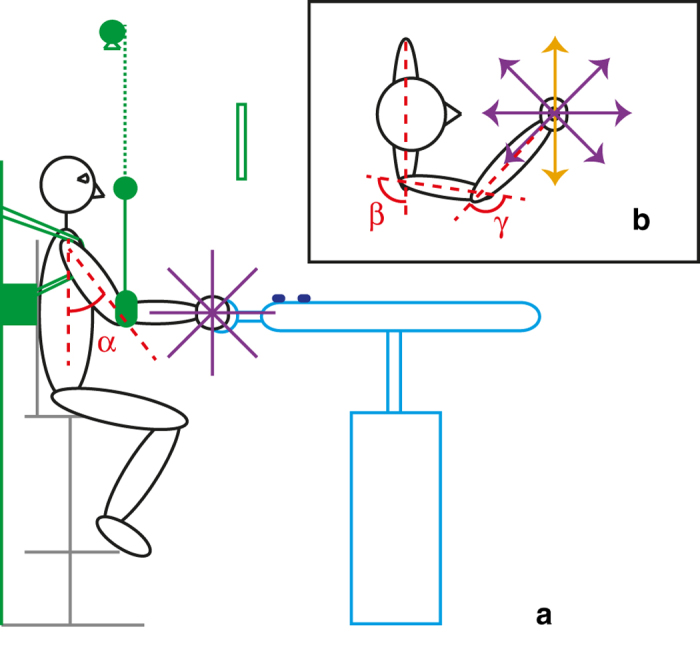
Schematic representation of the setup and the posture of the participants. The joint angles were the same for all participants. (**a**) Side view of the setup. In green, the back and top frame are drawn. The back frame was attached to the back of the chair, while the top frame was positioned above the participant. The back frame prevented movement of the chair, while the shoulder straps kept the participant in the same position throughout the experiment. A sling was attached to the top frame, in which the lower arm was inserted, to make sure that only small movements of the elbow in the horizontal plane were possible. Above the top frame, a webcam was mounted to check the posture of the participants. In grey, the chair is shown, which could be adjusted in height and position before it was attached to the back frame. The HapticMaster and the infrared Optotrak markers on top are drawn in light and dark blue, while the participant is shown in black. In purple, the four planes in which the 26 force directions were defined are shown. In experiment 1, participants were blindfolded. In experiment 2, participants received visual feedback via the screen (green open rectangle), which they could see through a gap in the frame (dashed green line). A cloth (green, underneath frame) prevented them from seeing their own hand. (**b**) Top view of the setup, showing the 8 force directions that were defined in each of the planes. The orange directions are the ones that are present in every plane, resulting in 4 × 6 + 2 = 26 unique directions. The red dotted lines and arcs show the postural angles: *α* = 38°, *β* = 98°, *γ* = 125°.

Figure 5 shows an overview of the experimental setup. Participants were seated on a height-adjustable chair. In experiment 1, they were blindfolded. In experiment 2, a black cloth attached to the frame in front of their head prevented them from seeing their hand, while they could still see the screen, which was used for visual feedback. Their torso was fixed to the back of the chair using shoulder straps. Participants were asked to hold the handle of a haptic device, the HapticMaster (Moog Inc.)^45^. To prevent a change of posture, elbow height was fixed using an elbow sling, which was attached to the frame. The height and position of the chair, the position of the frame and the position of the elbow sling were adjusted to ensure that all participants performed the experiment using the same posture, with a slightly flexed, abducted shoulder and a slightly flexed elbow. Webcams were placed above and at the right side of the setup to check the angles of the upper and lower arm. For all participants, the following angles were used: torso-upper arm 38° in side view (see Fig. 5a for this angle) and 98° in top view, upper arm-lower arm 125° in top view (see Fig. 5b for the latter two angles). The lower arm was positioned in the horizontal plane, so the height of the arm sling was the same as the height of the handle of the haptic device, whereas the central position of the handle of the device was always in front of the sternum of the participant.

During experiment 2, force and position data were recorded by the HapticMaster at a sampling rate of 256 Hz. The recorded force was the force exerted on the ball-shaped handle (diameter 42 mm) of the haptic device. The recorded position was the position of the center of the handle of the device, which was rigidly connected to the device with a metal bar. During experiment 2, positions were also recorded using two infrared Optotrak markers, positioned on the horizontal tube of the HapticMaster (see Fig. 5a). These data were also collected with a frequency of 256 Hz. For the analysis, the position data measured with the Optotrak markers were used, as they did record position at endpoint level and therefore most closely resembled the true position of the handle. A comparison of the position data of the HapticMaster and those of the Optotrak revealed that both systems yielded the same results for frequencies below 10 Hz, which were the frequencies of interest for this experiment. However, our method was based on the method described by Trumbower *et al.*^25^, in which Optotrak position data were used. For consistency, the same was done in our experiment.

### Procedure

#### Experiment 1: Perception

During experiment 1, participants were presented with a force (2, 3, 4, 5, or 6 N) in one of the 4 planes shown in Fig. 5a. In each plane forces were presented in 8 directions, as shown in Fig. 5B. This resulted in 26 unique predefined directions, in which forces were exerted on the participant’s right hand using the handle of a HapticMaster. The force was increased from zero to the test force in two seconds using a linear force ramp, which thus had a different slope for every force level. Participants could therefore not use the duration of the force ramp as a cue to estimate the force level. When the force reached the test level, a tone indicated that participants could verbally indicate the magnitude of the force. They could use a number on any scale, as long as they kept their scale linear and used the same scale throughout each session. There was no time limit, but participants generally answered within a few seconds after the tone. When an answer was given, the force was ramped down in 1.5 seconds to zero again. After this, a relax phase of three seconds followed, after which the next trial started. During the force presentation, participants were instructed to keep their hand steady. If they moved their hand more than 3 cm from the starting point in any direction, a tone indicated they had exceeded the limits. If this happened, the trial was rejected and the handle moved back to the start position to initiate a new trial. All rejected trials were repeated at the end.

Experiment 1 consisted of 650 trials per participant, divided over 3 one-hour sessions. All direction-magnitude combinations were repeated 5 times. The order of combinations was pseudo-randomized, by making sure that participants first experienced all possible combinations once in a random order, after which all possible combinations were presented a second time in random order, etc. To avoid fatigue, each session was divided into 4 blocks of 52 to 55 trials, which took about 10 min per block. In between the blocks, participants were asked to relax their arm and hand and take some rest to make sure no effect of fatigue was present. They indicated when they were ready to proceed to the next block themselves. Participants were given some practice trials before starting the measurements. In these practice trials, they were given no feedback on their performance other than technical instructions on how to perform the task as it was intended.

#### Experiment 2: Arm dynamics

In experiment 2, arm dynamics were measured in the 26 directions in which force perception was measured in experiment 1. Each trial started similar to those in experiment 1: a force of 4 N was ramped up linearly and participants were instructed to resist this force, resulting in a steady hand position. Eight seconds after reaching the plateau level of the constant force, 35 seconds of stochastic position perturbations started (see below for a description of the signal design). Participants were instructed to keep exerting the constant force of 4 N (which was always in the same direction as the perturbations), while not intervening with the perturbations in any way. By asking participants to keep exerting the force, the voluntary force level in experiment 2 was kept as comparable to that in experiment 1. The participants were aided in this task by feedback on a screen that showed the force target, which was the force ramp followed by the force plateau of 4 N. On top of that, the filtered measured force along the perturbation direction was plotted, using a running average filter with a window size of 94 ms. It was stressed to the participants that their task was only to keep the mean force at 4 N and not to intervene with fluctuations in the force signal around this mean force, which were induced by the perturbations.

Experiment 2 consisted of 26 trials, presented in a random order. Each perturbation signal lasted 35 seconds, resulting in a total time of ~50 seconds per trial. Between trials, participants could relax for a few seconds. There was a large break to avoid fatigue, dividing the experiment in two blocks of about 15 minutes each. Again, participants could indicate themselves when they felt ready to proceed to the next block. Participants were given some practice trials before starting the measurements. In these practice trials, they were given no feedback on their performance other than technical instructions on how to perform the task as it was intended.

### Position disturbance signal design

The position disturbance signals were designed as a one-dimensional version of the method described by Trumbower *et al.*^25^. For every trial, a white noise signal of 35 seconds was generated, that was filtered using a second-order Butterworth filter with a cut-off frequency of 4 Hz. The signal was scaled to an RMS amplitude of 5 mm, resulting in a maximum movement amplitude of about 20 mm. For an example of a section of such a signal, see Fig. 3a. The position signal was used as the input signal for the HapticMaster, which was programmed as a position perturbation system by using stiff (20 kN/m) springs, with a damping ratio of 0.3, to send the handle to the required positions.

For the arm dynamics measurements, a one-dimensional approach was taken, because it most closely resembled the situation in which perception was measured. For measuring perception, one force direction per trial was used, which is congruent to one-dimensional perturbations in the arm dynamics measurements. To make sure that the level and direction of voluntary force was the same during the arm dynamics measurements as during the perceptual measurements, the approach of asking participants to exert a bias force (‘keep exerting this 4 N’) on top of a relax task (‘do not intervene with the perturbations’) was necessary (see Mugge *et al.*^38^ for a description of the influence of different task instructions on arm dynamics). We could have chosen to perform 3D perturbations for every bias force direction, which would have resulted in 26 slightly different ellipsoids per parameter and participant, which would all have been slight variations of the ellipsoids that could have been measured in a relax task^36^. However, we still would have only used one data point per ellipsoid in the analysis: the data point in the direction of the bias force. So, in our one-dimensional approach, we did not measure 26 data points on 1 ellipsoid, but gathered data of one specific direction of 26 ellipsoids per participant, which was sufficient to answer our research question.

### Data analysis

#### Perceptual data

The mean of the magnitude perception data per session was scaled to the actual mean force of that session, which was always very close to 4 N (the precise value depended on the order of the magnitude-direction combinations). This was done to correct for the fact that participants might have changed their scale between sessions. A power function was fitted to describe the relation between physical force magnitude and perceived force magnitude for the complete data set, as shown in Fig. 1. Subsequently, the mean perceived force magnitude values per physical force magnitude were calculated according to the fit. By dividing the actually perceived force magnitude values by these calculated mean values, the data were normalized. The normalized data were used in the further statistical analyses.

#### Arm dynamics data

Since position perturbations were used to measure arm impedance, an open-loop approach could be used for the analysis of the arm dynamics data (as shown in de Vlugt *et al.*^19^ and Perreault *et al.*^18^). For each trial, the registered force and position data (for examples, see Fig. 3a,b) were analyzed in the frequency domain, by first calculating the spectra of both signals using a Fast Fourier Transform (for examples, see Fig. 3c,d), from which cross-spectral densities could by determined. The first data point (at 0 Hz) was removed in all spectra to eliminate the constant force from the analysis. To obtain smoother cross-spectral densities and to obtain a better estimate of the coherence of the signals, averaging over 4 adjacent frequencies was used^46^. From these averaged cross-spectral densities, transfer functions (also called Frequency Response Functions or FRFs) and their coherence were calculated. The transfer functions were calculated in this way:


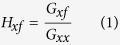


with *H*_*xf*_ being the FRF for that trial, as illustrated in Fig. 3f, *G*_*xf*_ being the cross-spectral density of the position and force signal and *G*_*xx*_ being the power spectral density of the position signal. To assess the linearity of this response function and the amount of noise in the system, coherence (see Fig. 3e) was estimated using:


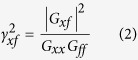


For a more elaborate description of the derivation of Equations 1, 2 and the averaging method, see de Vlugt *et al.*^19^.

#### Impedance model fit

One of the most simple ways to model the human arm is by describing it as a mass-damper-spring system, of which the general formula in the time domain is:


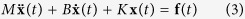


with *M*, *B* and *K* being the inertia, damping and stiffness parameters describing the system, **f** being the force and **x** being the position. In the frequency domain, this function can be written as:


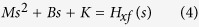


in which *H*_*xf*_ is the FRF, 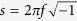 and *f* is the frequency. This model has proven to be a representative model of a human arm for small displacements, when participants are asked not to intervene with the perturbations, such as in the present study^3^,^14^. Since we estimated the FRFs from our measured data, as shown in Equation 1, we could fit the model in Equation 4 to these FRFs to obtain the system parameters. The model was only fitted for frequencies below 10 Hz, since visual inspection of the FRFs and a decline in the coherences showed that the system started deviating from a second-order system above this frequency. Probably, contact dynamics started playing a role above 10 Hz^38^, in which we were not interested. In these types of experiments, fits are commonly restricted to frequencies below 10 Hz^25^,^27^,^36^. The fitting was performed using a logarithmic least squares fitting procedure in which the following error was minimized:


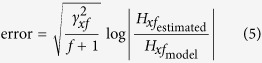


with 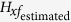 being the FRF estimated from the measured data and 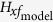 being the FRF resulting from the model in Equation 4, when using the fit parameters *M*, *B* and *K*. The coherence of the measured signal 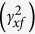 and the frequency ( *f* ) were used as weight factors, to make sure that low-coherent data points receive less weight and to compensate for the fact that at higher frequencies the data points are more tightly spaced in a logarithmic representation. An example of such a fit is shown in Fig. 3f. By performing this fit for all the FRFs in the 26 measured directions, 26 values for stiffness, damping and inertia were found per participant.

#### Statistics

To assess the effect of direction, a repeated measures ANOVA with direction as the within-subject variable was performed on the parameter perceived force magnitude for experiment 1 and on the parameters stiffness, damping, and inertia for experiment 2. For the analysis of experiment 1, the interaction between the effect of force direction and the variables physical force and session was also tested. When the sphericity criterion was violated, Greenhouse Geisser correction was used.

To obtain a description of the orientation of the data, a principal component analysis (PCA) was performed on the 26 values found for perceived force magnitude, stiffness, damping, and inertia, for each participant. This analysis describes data using three orthogonal axes for three dimensional data, by aligning the axes with the directions with the largest amount of variance in the data. Therefore, the first component of the PCA shows the major axis of the data, while the third component reveals the minor axis. From these components, the eccentricity was calculated, which is the ratio between the minor and major axis. The similarity of the orientations of the perceptual data set and the data sets of each of the arm dynamics parameter was assessed by testing the similarity in major axis direction. To obtain one value per vector describing the orientation, all vectors of each combination of perceptual data and one of the arm dynamics parameters were projected on a 2D plane. This 2D plane was defined as the plane spanned by the mean vector of the perceptual data and the mean vector of the arm dynamics parameter of interest.

When a test was needed to assess if the values in a data set differed from 1, a one-sample *t*-test was used. When the means of two independent data sets had to be compared, an independent samples *t*-test was used, with Bonferroni correction when appropriate. When equal variances could not be assumed, the degrees of freedom and *p*-value were adjusted accordingly. For all statistical analyses, *p* < 0.05 was deemed significant.

## Additional Information

**How to cite this article**: Beek, F. E. *et al.* Haptic perception of force magnitude and its relation to postural arm dynamics in 3D. *Sci. Rep.* **5**, 18004; doi: 10.1038/srep18004 (2015).
